# Medical Society of the County of Chautauqua

**Published:** 1895-09

**Authors:** 


### Society Proceedings.

### MEDICAL SOCIETY OF THE COUNTY OF CHAUTAUQUA.

THE society held its annual meeting at the Thompson House, Mayville, Tuesday, July 11, 1895, at 11 o'clock A. M. Officers for the ensuing term were elected as follows: president, E. S. Rich, M. D., of Kennedy; vice-president, Morris N. Bemus, M. D., of Jamestown; secretary-treasurer, C. A. Ellis, M. D., of Sherman; censors, James Murphy, M. D., of Sherman, Thomas D. Strong, M. D., of Westfield, and A. H. Bowers, M. D., of Jamestown.

Reports and discussions occupied the remainder of the morning session when the meeting adjourned to the Sherman House, Jamestown, the second Tuesday in January, 1896. Dinner was served at the Thompson House.

The society took the early afternoon steamer for Lakewood where a scientific session was held at the Kent House. The following papers were read: President's address, Dr. Nelson G. Richmond, Fredonia; The Heart in Anemia, Dr. De Lancey Rochester, Buffalo, N. Y.; Abnormalities of the Urine in Disease and their Significance, Dr. Edward C. Lyman, Jamestown; Some Observations in the Treatment of Cerebro-spinal Meningitis, Dr. E. M. Scofield, Jamestown.

Dr. C. E. Lundgren, of Jamestown, had a paper on The Abuse of Some Narcotics, but there was not time for all and he withheld the paper for the next meeting.

Dr. James T. Whittaker, of Cincinnati, a guest at the Kent House, was invited to attend the meeting. He gave important information regarding the use of antitoxin. He was the first to use antitoxin in this country.

Supper was had at the Kent, after which the members and guests went to Jamestown.

Those in attendance were Drs. Thomas D. Strong, W. A. Putnam, Westfield; E. S. Rich, Kennedy; C. A. Ellis, James Murphy, Sherman; V. M. Griswold, Fredonia; William Duke, Lily Dale; E. A. Scofield, Bemus Point; George F. Smith, Sinclairville; J. C. Lewis, Panama; William M. Bemus, Morris N. Bemus, A. H. Bowers, C. E. Lundgren, E. M. Scofield and E. C. Lyman, Jamestown. Dr. De Lancey Rochester, of Buffalo, was a guest of the society.

---

Treatment of Early Abortion.—A conservative treatment of early abortion is recommended by Schauta, of Vienna, in a recent article. The old rule that pain and hemorrhage combined mean inevitable abortion he does not indorse, and thinks the accident preventable as long as the hemorrhage is not excessive or the cervix dilated. He does not try to check the bleeding and trusts to rest, which is continued for eight days after the last bleeding. In those cases where the os dilates and abortion becomes inevitable, he tampons with a strip of iodoform gauze about two yards long and the width of three or four fingers' breadth. He prefers to retract the perineum with the fingers of the other hand instead of using the speculum, and renews the tampon at the end of twenty-four hours at the least, removing it sooner if the appearance of sacral pains indicates that the ovum has been expelled from the womb. This method of treatment is in sharp contrast to the radical advice so often given of late to go in at once and curette out the womb as soon as an abortion beeomes inevitable—a practice which Schauta objects to because of the danger of leaving fragments of membrane behind.—*Western Lancet.—Medical and Surgical Reporter.*

